# Assessing awareness of colorectal cancer symptoms: Measure development and results from a population survey in the UK

**DOI:** 10.1186/1471-2407-11-366

**Published:** 2011-08-23

**Authors:** Emily Power, Alice Simon, Dorota Juszczyk, Sara Hiom, Jane Wardle

**Affiliations:** 1 Cancer Research UK, Angel Building, 407 St John Street, London, EC1V 4AD, UK; 2 Cancer Research UK Health Behaviour Research Centre, Department of Epidemiology and Public Health, University College London, Gower Street, London, WC1E 6BT, UK

## Abstract

**Background:**

This paper describes the development of a Cancer Awareness Measure for colorectal (CRC) cancer (Bowel/Colorectal CAM^a^) (study 1) and presents key results from a population-representative survey using the measure (study 2).

**Methods:**

**Study 1**

Items were taken from the literature and reviewed by expert groups. A series of three validation studies assessed reliability and validity of the measure. To establish test-retest reliability, 49 people over 50 years of age completed the Bowel/Colorectal CAM on two occasions (range 9-14 days, mean 13.5 days). Construct validity was assessed by comparing responses from bowel cancer experts (n = 16) and the lay public (n = 35). Lastly, a brief intervention study tested sensitivity to change with participants (n = 70) randomly allocated to be given a control leaflet or an intervention leaflet and their responses were compared.

**Study 2**

1520 respondents completed the Bowel/Colorectal CAM in a population survey carried out by TNS-British Market Research Bureau International (TNS-BMRB) in March 2010.

**Results:**

**Study 1**

Internal reliability (Cronbach's alpha = 0.84) was high. Test-retest reliability was over r = 0.7 for warning signs, risk factors and age people are first invited for screening, but lower (between 0.6 and 0.7) for other items (lifetime risk, awareness of bowel cancer screening, age at risk). Bowel cancer experts achieved higher scores than equally educated controls (54.7 [4.3] vs. 42.9 [5.7]; P < 0.001) demonstrating the measure has construct validity and intervention participants showed higher knowledge than controls (51.4 [5.9] vs. 42.9 [5.7]; P < 0.001) suggesting the measure is sensitive to change.

**Study 2**

Respondents recalled on average, one CRC sign and one risk factor. There was particularly low prompted awareness of the signs 'lump in the abdomen' (64%) and 'tiredness' (50%) and several lifestyle risk factors for CRC, e.g. exercise (37%).

Respondents from more affluent groups had consistently higher knowledge of signs and risk factors compared to those from more deprived groups.

**Conclusions:**

The Bowel/Colorectal CAM meets accepted psychometric criteria for reliability and construct validity and should therefore provide a useful tool for assessment of CRC awareness. The population survey revealed low awareness of several CRC signs and risk factors and emphasises the importance of continuing public education, particularly about the link between lifestyle behaviours and CRC.

## Background

Colorectal cancer (CRC) is the third most common cancer in the UK with over 38,000 people diagnosed each year [1]. In 2008, 16,259 people died from the disease, making it the second largest cause of cancer death [1]. Men have a lifetime risk of 1 in 16 compared with 1 in 20 for women and incidence rates are substantially higher in socioeconomically deprived groups [2].

The average five-year survival for CRC in England is 51%, but survival rates vary by disease stage at diagnosis, with over 93% of patients surviving a diagnosis of localised disease (Dukes stage A) compared with just 7% of those with distant metastases [3]. Currently, only around 13% of cases are diagnosed at an early stage. Furthermore, survival is significantly poorer in England than the European average (1 year survival: 72% vs 76%) [4]. Achieving earlier diagnosis should play a role in reducing this difference [4].

Early diagnosis of CRC can be improved by ensuring high participation in available screening programmes such as the National Bowel Cancer Screening Programme (NBCSP) in England, Wales and Scotland, which offers Faecal Occult Blood Testing (FOBt) every two years to people aged 60-74 (varying by country), with abnormal results followed up with colonoscopy. FOBT screening was recently estimated to achieve a 15% reduction in mortality [5], but participation in CRC screening in the UK, as well as worldwide, is relatively low [6].

Although screening is an important route for early diagnosis, it does not cover all cases or age groups, in addition, many cases of CRC will present with symptoms in primary care or elsewhere. The Department of Health in England recently issued a set of 'key messages' for bowel cancer which include signs and symptoms as well as risk factors associated with the disease http://tinyurl.com/6kfs6pl. Detectable symptoms include a persistent change in normal bowel habit, bleeding from the back passage, a lump in the abdomen and unexplained extreme tiredness. Modifiable risk factors include red and processed meat consumption, body fatness and alcohol consumption [7]. Studies of public awareness of symptoms and risk factors reveal that knowledge is poor [8-10]. For example, in a survey of adults in the UK, 24% could not correctly name any warning signs for CRC and over half (58%) were unable to correctly recall any risk factors [8]. Raising awareness of these, along with awareness of the NBCSP, could help reduce cases, ensure earlier detection and improve survival.

The Cancer Reform Strategy (CRS), published by the Department of Health in 2007, emphasised the importance of raising awareness of cancer early warning signs in the general population [11]. The National Awareness and Early Diagnosis Initiative (NAEDI), which is a partnership between Cancer Research UK (CR-UK) and the Department of Health, was set up as part of the CRS to support and co-ordinate activities that promote earlier diagnosis of cancer and ensure delivery of the CRS. An early output of NAEDI was the Cancer Research UK Cancer Awareness Measure (CAM), which is the first validated tool to assess awareness of generic cancer signs and risk factors, [12]. In the UK, the CAM is being used nationally and locally to benchmark (e.g. [13,14]) and track awareness over time, as well as to monitor the impact of initiatives intended to improve awareness of cancer.

Several ad-hoc scales have been devised to measure CRC cancer awareness [15,16], but they use different question formats (e.g. true/false questions vs. unprompted questions) making it difficult to compare across studies. A measure of CRC awareness that follows the format of the generic CAM will make it possible to establish current levels of awareness and evaluate awareness-raising initiatives using a standardised, validated instrument.

This paper describes the development of a Cancer Awareness Measure for colorectal cancer (Bowel/Colorectal CAM) (Study 1) and presents key results from a population-representative survey using the measure (Study 2).

## Study 1

### Methods

#### Item generation

Items were generated using the Department of Health's 'bowel cancer - key messages' [17] and the National Institute for Health and Clinical Excellence (NICE) 'Referral guidelines for suspected cancer' [18]. Additional warning signs and risk factors were identified from a review of the published and 'grey' literature and current public health material (e.g. CR-UK leaflets). The format used in the generic CAM (see [19] for further details) was used for most items.

Items were reviewed by experts (N = 16) in CRC (experts included a gastrointestinal and colorectal consultant oncologist, a colorectal surgeon, a medical oncologist, a surgeon consultant clinical oncologist, a lead consultant for general surgery, and a consultant general surgeon) who considered interpretability, clarity and accuracy. Draft versions of the measure were also used in 17 cognitive interviews with members of the public (aged 22-74 years). This 'think-aloud' method [20] explores respondents' comprehension of the questions, i.e. whether specific words and phrases used in the question are understood as intended by researchers and adjustments are made accordingly. For example, the warning sign 'straining feeling' was changed to 'a feeling that your bowel does not completely empty after using the lavatory'.

The final version of the Bowel/Colorectal CAM consisted of 27 questions, including 10 on awareness of warning signs (1 unprompted and 9 prompted), 11 on risk factors (1 unprompted and 10 prompted), one on delay in help-seeking, one on age at risk, one on lifetime risk of CRC, two on awareness of UK CRC screening programmes and one question on confidence in detecting a CRC symptom. Awareness of warning signs and risk factors were assessed with two items each. The first item was unprompted and asked respondents to generate responses from memory. The second item (prompted) presented a list of symptoms or risk factors and asked respondents to indicate whether they could be symptoms of CRC with response options 'Yes'/'No'/'Don't know' for warning signs and a 5-point likert agreement scale for risk factors ('Strongly agree' - 'Strongly disagree'). All the Cancer Awareness Measures, including the Bowel/Colorectal CAM are available to download from the Cancer Research UK website [21].

#### Item scoring

For unprompted items assessing knowledge of warning signs and risk factors, a score of 1 was given for each sign or risk factor mentioned that also appeared in the prompted item lists. Scores were added together to give total scores for unprompted awareness of signs (maximum score of 9) and unprompted awareness of risk factors (maximum score of 10). For the prompted warning sign items, 'No' and 'Don't Know' responses were combined and scored '0' and 'Yes' responses were scored '1'. For the prompted risk factor items 'Strongly agree' or 'Agree' responses were scored '1' and 'Not sure', 'Disagree' and 'Strongly disagree' responses were scored '0'. Total scores for prompted awareness of warning signs (maximum score of 9) and risk factors (maximum score of 10) were calculated by adding the recoded responses together.

The 'delay in help-seeking' item was scored from 1-10 ('1-3 days' - 'Never') (see 'Additional File 1 - help-seeking item'). For the 'age at risk' item 'a 60 year old' response was scored '1' and all other responses were given a score of '0'. The question on lifetime risk of CRC was recorded verbatim and recoded to reflect accuracy of response with '5' or '6' (out of 100) given a score of '1' (accurate) and all other estimates scored '0' (inaccurate) (see Additional File 2 - lifetime risk item). For the item measuring awareness of the NHS Bowel Cancer Screening Programme 'No' and 'Don't know' responses were combined and scored '0' and 'Yes' responses were scored '1'. Responses to the item asking what age people were first invited to screening were recorded verbatim and recoded so that 'at 60 years' was scored '1' (accurate) and all other responses were scored '0' (inaccurate). The 'confidence in detecting a symptom' item was scored from 1-4 ('Not at all confident' - 'Very confident').

A 'total knowledge score' was calculated by adding the total score for prompted warning signs and risk factors to scores for items: age at risk of CRC, lifetime risk, awareness of the NHS Bowel Cancer Screening Programme and the age people are first invited.

#### Participants

##### Sample 1

49 people recruited from four 50-plus community centres across three London boroughs (Westminster, Hackney, Kensington and Chelsea) completed supervised paper-and-pencil versions of the CAM on two occasions approximately two weeks apart. Test-retest reliability assesses the consistency of a measure over time and is therefore dependent on the stability of the measured construct, in this case, awareness of CRC. It was therefore important to leave adequate time to ensure that respondents did not recall their previous responses to the Bowel/Colorectal CAM, while minimising the likelihood that their knowledge about CRC changed during the intervening period. Participants were aged between 54 and 91 (mean 69 years), 90% were from a White ethnic background, most (71%) were retired, and 20% had no formal qualifications. They were paid £5 for completing the questionnaire on two occasions.

##### Sample 2

70 University College London administrative staff volunteered to complete an online version of the CAM in response to an email about the study. Half received CR-UK's educational leaflet (n = 35) ('Bowel Cancer: The Facts'), while the others received a control leaflet (n = 35) ('Recycle to Save the Environment').

##### Sample 3

16 experts on bowel cancer from charities committed to raising awareness of CRC ('Beating Bowel Cancer' and 'Bowel Cancer UK') completed an online version of the questionnaire. These experts were different from those that helped to generate items and review the Bowel/Colorectal CAM.

Participant data was anonymised at all times and therefore this research was exempt from ethical approval.

#### Analyses

Validation of the Bowel/Colorectal CAM included analysis of test-retest reliability using Pearson's correlation; internal reliability was assessed using Cronbach's alpha; specific item analyses (item difficulty and item discrimination analyses) were analysed by examining simple descriptive statistics (e.g. percentages) and construct validity and sensitivity to change were analysed using Chi-Square and t-tests.

## Results

### Reliability

Test-retest reliability was assessed using data from sample 1 (*n *= 49) who completed the measure twice over an interval of approximately 14 days (range 9-14 days; mean 13.5 days). Test-retest reliability was high for prompted warning signs (r = 0.77, P < .001); prompted risk factors (r = 0.78, P < .001)^b^; and age invited for screening (r = 0.71, P < .001), and moderate for lifetime risk (r = 0.67, P < .001); age at risk (r = 0.60, P < .001), awareness of the CRC screening programme (r = 0.65, P < .001) and total knowledge score (r = 0.69, P < .001).

Samples 1 and 2 were combined to assess internal reliability (*n *= 119). Two items were excluded, one on 'delay in help-seeking' and one on 'confidence in detecting a CRC symptom' because they did not relate to awareness or knowledge of CRC but instead provided an indication of perceived behavioural response in relation to symptom detection (delay in help-seeking) and perceived self-efficacy (confidence in detecting a CRC symptom). When using Cronbach's alpha, a minimum score of 0.7 is needed for a questionnaire to be considered reliable [22] and the results were good, with a Cronbach's alpha of 0.84 for the whole questionnaire, 0.73 for prompted warning signs and 0.79 for the prompted risk factors subscale.

### Item discrimination and difficulty

A highly discriminating item indicates that the participants who had high awareness scores answered that item correctly whereas those with low awareness scores answered it incorrectly. Useful items have an item-to-total correlation greater than 0.2 [23]. Combining results from Samples 1 and 2 (n = 119), all items met this criterion except for recognition of the symptom 'blood in stools'. As this item is part of the Department of Health's key messages for bowel cancer [17], it was considered important in terms of face validity and retained.

Item difficulty was assessed using results from Samples 1 (*n *= 49) and from the control group from Sample 2 (*n *= 35) on all items where it was possible to ascertain a 'correct' answer. Items should be retained if they are answered correctly by more than 20% and fewer than 80% of respondents [24]. Most items met these criteria, with four exceptions: two warning signs received higher rates of correct answers (>80% correct) (rectal bleeding and blood in stools), and one risk factor (diabetes) and the item on lifetime risk received a lower percentage of correct answers (<20% correct), but these items were retained for reasons of face validity.

### Construct validity

To establish construct validity, the 'known-groups' method was used where responses from experts are compared with a lay sample. Construct validity is supported when a questionnaire can discriminate between a groups of individuals known to have different levels of knowledge [25]. Sample 2 (control group only, *n *= 35) and Sample 3 (CRC experts, *n=*16) were used in these analyses. There were no significant differences in age, gender, ethnicity, educational qualifications or employment status between the groups, although participants from the 'lay' group were more likely to be married. CRC experts scored consistently higher than the 'lay' sample on all sections of the CAM (see Table 1).

### Sensitivity to change

A brief educational intervention was carried out to test whether the CAM was sensitive to increases in knowledge. If the measure is sensitive to change, those given CRC educational materials should score higher than those given control materials. Participants from Sample 2 (*n *= 70) were assigned to receive either a CRC information leaflet ('Bowel cancer: The Facts') or a leaflet about recycling ('Recycle to Save the Environment'). The two groups did not differ significantly in age, gender, ethnic background, marital status, employment status or educational qualifications. The CRC information group scored significantly higher than the control group on all awareness sections of the CAM (see Table 1).

**Table 1:** Bowel/Colorectal CAM construct validity and sensitivity to change

	Control (n = 35)	Expert (n = 16)	Intervention (n = 35)
**Demographics**		*N (%)*	

Age			

18-34	10 (31.2)	5 (31.4)	12 (41.4)

35-54	16 (50.0)	9 (56.3)	15 (51.7)

55+	6 (18.7)	0 (0)	2 (6.9)

Female	18 (51.4)	11 (68.7)	19 (54.3)

White ethnicity	23 (65.7)	12 (75.0)	25 (71.4)

Married/civil partner	20 (57.1)	1 (6.3)***	17 (48.6)

Employed full time	31 (88.6)	12 (75.0)	30 (85.7)

Degree or higher	23 (65.6)	11 (68.8)	24 (68.6)

**Knowledge**		*Mean (SD)*	

Warning signs (unprompted recall)	1.4 (1.3)	4.9 (1.5)***	3.6 (1.3)***

Warning signs (prompted)	5.9 (2.1)	8.1 (0.9)***	7.7 (1.2)***

Risk factors (unprompted recall)	0.97 (1.2)	3.5 (1.9)***	4.05 (1.6)***

Risk factors (prompted)	35.9 (3.7)	43.1 (3.7)***	40.8 (5.5)***

		*N (%)*	

Age at risk^a^	16 (45.7)	14 (87.5)**	30 (85.7)***

Bowel Cancer Screening Programme^a^	11 (31.4)	15 (93.8)***	31 (88.6)***

Age people first invited for screening^a^	4 (11.4)	14 (87.5)***	23 (65.7)***

Lifetime risk^a^	8 (22.9)	12 (75.0)***	18 (51.4)*

		*Mean*	

Total knowledge^b^	42.9 (5.7)	54.7 (4.3)***	51.4 (5.9)***

Note. Not all demographics add up to 100% due to missing data.

Note. Construct validity was assessed by comparing the 'expert' group with 'controls' and sensitivity to change was assessed by comparing the 'intervention' group with the 'controls'

^a ^Shows those responding 'correctly'

^b ^Total knowledge score' = Warning signs (prompted) + risk factors (prompted) +age at risk + bowel screening programme + age people first invited for screening + lifetime risk

* indicates a statistically significant difference (P < 0.05) compared with control

** indicates a statistically significant difference (P < 0.01) compared with control

*** indicates a statistically significant difference (P < 0.001) compared with control

## Study 1 discussion

The Bowel/Colorectal CAM is a reliable and valid measure of CRC awareness, meeting standard psychometric criteria. Reliability was high, with a total Cronbach's alpha of 0.84 and all subscales above the recommended level of ≥ 0.7 [21] and it had test-retest reliability over the recommended r = 0.7 [24] for warning signs, risk factors and the item on age people are first invited for screening. Slightly lower correlations (between 0.6 and 0.7) were obtained for some items (lifetime risk, awareness of bowel cancer screening, age at risk and the total Bowel/Colorectal CAM score). This may indicate low awareness of these issues which resulted in respondents guessing their answers. These items were retained because they were considered to measure important components of CRC knowledge, but could be removed should a shortened version of the measure be needed. The measure was able to distinguish between different levels of CRC knowledge with experts scoring higher than an equally educated comparison group. This was further supported by the results of a brief intervention in which the measure was sensitive to increases in awareness of CRC.

Validation of the measure had some limitations which should be noted. Different modes of administration (pen-and-paper and online) were used in the validation studies, but mode of administration was consistent within each sub-study. Using an unsupervised online version of the measure to assess construct validity and sensitivity to change was not ideal, although we had ensured that participants could not go back to previous questions. Lastly, although reliability was assessed with an older-adult sample, reflecting the increased risk of CRC with age and the greater need for raised awareness in this group, the other validation studies used well-educated, predominantly White samples and in some cases, had small sample sizes, therefore findings may lack generalisability.

Overall, the findings from study 1 demonstrate that the Bowel/Colorectal CAM meets accepted psychometric criteria for reliability and construct validity. It should provide a useful tool for assessment of CRC awareness, evaluating the effectiveness of education campaigns designed to improve awareness, and making comparisons across studies.

Since the Bowel/Colorectal CAM was validated, a review of all the CAMs was undertaken, taking account of extensive feedback from local partners (Primary Care Trusts and Cancer Networks) who had used the measure(s). As a result of this review process, the item on lifetime risk (see 'additional file 2 - lifetime risk item') was removed from the Bowel/Colorectal CAM. As our validation studies had indicated, this item was proving too difficult for people to answer and produced data of limited value. In addition, the 10-item response scale for the help-seeking item ('1-3 days' - 'Never') was removed from the CAMs (including the Bowel/Colorectal CAM) to reduce the possibility that providing prompted options encouraged 'socially desirable' answers (leading to positively skewed data) (see 'additional file 1 - help-seeking item').

## Study 2

### Method

1520 respondents completed the Bowel/Colorectal CAM as part of a population survey carried out by TNS-BMRB in March 2010. The TNS-BMRB Omnibus survey uses random location sampling to obtain a population-representative sample of Great Britain. Respondents are drawn from a small set of homogenous streets, selected with a probability proportional to the population after stratification by Acorn characteristics (a geo-demographic segmentation tool) and region. The sampling frame is an amalgamation of Output Areas (used in the Census) which on average contain 300 households. They are stratified by region and within region by Acorn type, County and Independent Television (ITV) region. Quotas are set in terms of characteristics known to have a bearing on individuals' probabilities of being at home and therefore available for interview (age, working status). Respondents completed the CAM using a computer-assisted personal interview (CAPI) in their own homes and in the presence of trained interviewer. Respondent's data were anonymised at all times and therefore this research was exempt from ethical approval.

### Socio-demographic characteristics

The Omnibus survey includes several socio-demographic questions, of which the following were used in these analyses: gender; age (dichotomised into <50 and ≥50 years^c^), ethnicity (because of small numbers in each ethnic sub-group, ethnicity was categorised as 'White' vs. 'non-White'), marital status (married/cohabiting, not married); highest educational qualification (degree or above, below degree, other, no formal qualifications), and Social Economic Group (SEG) (AB = higher/intermediate managerial, administrative or professional; C1 = supervisory or clerical, junior managerial, administrative or professional, C2 = skilled manual workers, D = semi and unskilled manual workers, E = state pensioners or widows (no other earner), casual or lowest grade workers).

### Analyses

Data were analysed using SPSS 14.0. Data were weighted to ensure that the demographic profile of the sample matched that for all adults in Great Britain aged 16 or over (using Census data). Response rates to the Omnibus survey are not recorded by TNS-BMRB and therefore it was not possible to analyse differences between responders and non-responders. Chi-square tests were used to examine differences in CRC awareness (prompted) in relation to gender, age, ethnicity, SEG and familiarity with cancer. Multivariate analyses using General Linear Models assessed whether any observed differences in knowledge (prompted) by socio-demographic factors were independent.

## Results

During the interviews, respondent were also asked if they had ever been diagnosed with cancer, those that said they had (n = 86) were excluded from the sample. The resulting sample demographics are shown in Table 2 and approximate key population estimates although the sample included a larger proportion of respondents from non-White ethnic groups (12% vs. the census figure of 7.9%) [26].

**Table 2:** Demographic profile of population representative survey using the Bowel/Colorectal CAM

	Survey sample
	**N (%)**

*Gender*	
Male	703 (49.0)
Female	731 (51.0)
	
*Age*	
16-49	858 (59.8)
50 and over	576 (40.2)
	
*Social Economic Group (SEG)*	
AB	383 (26.7)
C1	423 (29.5)
C2	303 (21.1)
D	214 (14.9)
E	112 (7.8)
	
*Marital status*	
Married/co-habiting	857 (59.7)
Not married	577 (40.2)
	
*Education*	
Degree or higher	368 (25.6)
Below degree	743 (51.8)
Other	58 (4.1)
No formal qualifications	253 (17.6)
Missing	12 (0.8)
	
*Ethnicity*	
White	1334 (87.8)
Other	186 (12.2)

### Awareness of warning signs and symptoms (see Table 3)

**Table 3:** Awareness of CRC warning signs and symptoms (unprompted and prompted)

Sign/symptom	Unprompted % (n)	Prompted % (n)
Change in bowel habit	23.1 (331)	78.1 (1104)
Blood in stools	15.4 (221)	88.6 (1253)
Bleeding back passage	8.7 (124)	86.4 (1220)
Unexplained weight loss	4.1 (58)	74.9 (1058)
Pain in back passage	3.3 (47)	76.4 (1080)
Tiredness	2.0 (28)	50.1 (707)
Pain in abdomen	0.3 (4)	76.7 (1084)
Bowel does not empty	0.1 (1)	47.3 (668)
Lump in abdomen	0 (0)	64.4 (909)

Awareness of signs and symptoms was measured with two items, unprompted and prompted (see Study 1 Methods section for more detail).

Unprompted awareness of warning signs and symptoms of CRC was very poor, with average recall of less than one sign or symptom (mean: 0.7; SD. 0.9). The most well-recalled warning sign was 'change in bowel habit' mentioned by 23%, followed by 'blood in stools' (15%). Fewer than 10% of respondents mentioned any other sign and a quarter (25%) said they did not know any signs or symptoms.

Prompted awareness was higher than for unprompted with over 78% agreeing that a change in bowel habit, bleeding from the back passage, or blood in stools could be signs of bowel cancer. Less well-recognised signs included 'bowel does not empty' (47%), 'tiredness' (50%) and lump in abdomen (64%).

Women showed higher knowledge of signs and symptoms than men, they were more likely to recognise a change in bowel habit (83% vs 72%; p =< 0.001), 'bleeding from back passage; (89% vs 83%; p = 0.004), 'blood in stools' (92% vs 85%; P < 0.001), 'pain in back passage' (80% vs 73%; p = 0.004), 'tiredness' (54% vs 46%; p = 0.013, and 'unexplained weight loss' (79% vs 70%; P < 0.001). Older respondents were also better at recognising signs or symptoms. For example, 81% of those aged 50 or older recognised that unexplained weight loss could be a sign compared with 71% of those younger than 50 years.

Respondents from a White ethnic background showing higher recognition of several signs and symptoms compared with those from a non-White ethnic background including: 'change in bowel habit' (81% vs 64%; P < 0.001), 'pain in abdomen' (79% vs 66%; P < 0.001), 'bleeding from back passage' (89% vs 74%; P < 0.001), 'blood in stools' (91% vs 75%; P < 0.001), 'pain in back passage' (80% vs 60%; P < 0.001), and 'unexplained weight loss' (77% vs 63%; P < 0.001).

There were also socio-economic differences, with those from a higher SEG's more likely to recognise 'change in bowel habit' (86% vs 67%; P < 0.001), 'blood in stools' (93% vs 86%; p = 0.001) and 'tiredness' (60% vs 49%; P < 0.001) than those from a lower SEG.

Knowing someone who had been diagnosed with cancer was associated with higher awareness. These respondents more likely to recognise several of the signs and symptoms of bowel cancer compared with respondents who did not know anyone with cancer (e.g. 'blood in your stools' 91% vs 75%; P < 0.001).

In multivariate analyses gender (P < 0.001), ethnicity (P < 0.001), SEG (P < 0.001) and familiarity with cancer (P < 0.001) were significant predictors of total (prompted) symptom score.

### Awareness of risk factors (see Table 4)

**Table 4:** Awareness of CRC risk factors (unprompted and prompted)

Risk factor	Unprompted % (n)	Prompted % (n)
Close relative with CRC	19.5 (280)	65.3 (909)
Drinking alcohol	18.6 (266)	46.1 (636)
Low physical activity	8.7 (124)	36.9 (508)
Overweight	2.8 (41)	59.1 (814)
Older age	2.7 (38)	45.3 (629)
Low fibre	2.1 (31)	61.0 (844)
Red and processed meat	0.8 (12)	41.1 (566)
Low fruit & vegetables	0.8 (12)	47.4 (663)
Bowel disease	0.4 (6)	61.9 (848)
Diabetes	0.1 (1)	25.8 (345)

Awareness of risk factors was also assessed using an unprompted and prompted question. Unprompted awareness of risk factors was low with average total recall of just over one (mean: 1.4; SD. 1.4). The most well-recalled risk factor was having a close relative with the disease, mentioned by 20%, followed by alcohol (19%). Fewer than 10% of respondents named any other risk factor and 28% said they could not name any.

Prompted awareness was highest for having a close relative with bowel cancer (65%) and bowel disease (62%). But less than half the respondents thought that having less than five portions of fruit and vegetables and drinking more than one unit of alcohol could increase the risk of bowel cancer (47% and 46% respectively). Less well-recognised risk factors included having diabetes (26%), low levels of physical activity (37%) and high red or processed meat consumption (41%).

Men were more likely to know about links between lifestyle behaviours and CRC than women; for example 64% agreed that being overweight could be a risk factor compared with 55% of women, and 42% thought a lower level of physical activity was associated with increased bowel cancer risk compared with 33% of women. Older respondents were more likely to know that low fibre could increase the risk of bowel cancer (67% vs 57%; P < 0.001), but those younger than 50 were more aware of the risks associated with drinking alcohol (49% vs 41%; p = 0.009) and older age (49% vs 40%; p = 0.004).

Although respondents from a non-White background were less likely overall to recall risk factors for bowel cancer, they were more likely to identify consumption of red and processed meat (50% vs 39%; p = 0.004), low-levels of physical activity (46% vs 35%; p = 0.001) and having diabetes (32% vs 24%; p = 0.018) as risk factors compared with White respondents.

The strongest differences were observed for SEG. Respondents from a higher social group more likely to know about several risk factors for bowel cancer compared to those from a lower social group (fruit and vegetables (63% vs 41%; P < 0.001), red and processed meat (50% vs 29%; P < 0.001), fibre (72% vs 56%; P < 0.001), overweight (66% vs 49%; p = 0.004), and exercise (44% vs 29%; p = 0.004).

In multivariate analyses ethnicity (P < 0.05) and SEG (P < 0.001) were significant predictors of total (prompted) risk factor score.

## Study 2 discussion

Awareness of signs and symptoms of CRC and risk factors was low overall with respondents in this population survey recalling on average, one CRC sign and one risk factor. There was particularly low awareness of lump in the abdomen and tiredness, both key messages for CRC http://tinyurl.com/6kfs6pl. Efforts to inform the public about detectable signs and symptoms of CRC are important, especially for symptoms that are more strongly associated with a diagnosis of CRC, (e.g. bleeding from the back passage) and particularly in older adults who are at most risk [27].

Awareness of several lifestyle factors associated with CRC (e.g. exercise, red and processed meat, alcohol and fruit and vegetable intake) was particularly poor. This has been found previously [8,9,28-30] and demonstrates that there is still a long way to go in educating the public about the association between living a healthy lifestyle and cancer risk. Public health initiatives to increase awareness of these risk factors are essential in ensuring better understanding of the links between lifestyle and cancer. Greater awareness could also lead to increased healthy behaviours [e.g. [28]] and thus could go some way towards reducing the overall burden of ill-health on the population.

Prompted awareness of CRC symptoms and risk factors was much higher than unprompted awareness, particularly for symptoms, where nearly half (47%) respondents recognised the CRC symptoms presented to them. This is in line with findings from surveys carried out using the generic CAM which also showed a much higher level of knowledge in response to prompted than unprompted CAM questions [13]. Further research should compare how unprompted and prompted knowledge are related to attitudes and help-seeking behaviour.

Women knew more CRC symptoms than men. This has been shown in other studies [e.g. [8,10,13]] and indicates that campaigns targeting men have yet to make an impact on the gap in knowledge between the sexes (e.g. 'Tackle it' by Beating Bowel Cancer). There were fewer gender differences in awareness of CRC risk factors, and men had higher awareness of the importance of weight and exercise. This could be because men are aware that lifestyle risk factors are more strongly associated with cancer in men than in women [31], or more likely reflects men's tendency to believe that cancer risk is modifiable [29].

Older respondents showed higher knowledge of symptoms for CRC than younger respondents which is encouraging because they are at higher risk and have a greater need to correctly identify symptoms. However, ironically, younger respondents were more aware of the link between CRC and older age than older respondents. Younger respondents also had higher awareness of alcohol and bowel disease as risk factors for CRC, which could reflect better knowledge of CRC risk factors among younger adults more generally.

Respondents from a White ethnic group had higher knowledge of several CRC symptoms but were less good at recognising risk factors than non-White groups. Respondents from a Non-White ethnic background are at lower risk of cancer overall and some groups (e.g. Asian) show lower incidence of some cancers including CRC [32]. Westernisation of diets, such as increased consumption of red meat could lead to increased CRC rates among these groups [e.g. [33,34]]. Non-White groups are less likely to participate in screening for CRC [6] and so it is important that interventions to raise awareness of CRC risk factors and symptoms target all ethnic groups.

Knowing someone with CRC was associated with higher awareness of CRC symptoms, but not risk factors. This is not that surprising given that increased exposure to CRC is likely to be associated with greater awareness of the disease and its presenting symptoms and less so with the causal processes involved. Having a family history of CRC could be more strongly associated with knowledge of risk factors due to increased perceptions of risk and motivations to prevent the disease [29].

More affluent groups showed higher awareness of some CRC symptoms and were consistently more knowledgeable about risk factors. This pattern is evident in other studies carried out in the UK and Europe [8,9,35,36] and is also apparent in awareness of cancer in general [e.g. [13]]. CRC incidence rates in men are 11% higher in the most deprived groups than affluent groups [2]. Raising awareness of CRC symptoms and risk factors and available screening programmes, particularly among men, is therefore imperative if we are to ensure that inequalities in CRC outcomes do not increase.

Certain limitations of this study should be noted. Response rate was not recorded by the survey company prohibiting a comparison of responders and non-responders, but data were weighted to ensure that the demographic profile of the sample matched that for all adults in Great Britain aged 16 or over (using Census data). It should also be acknowledged that the Bowel/Colorectal CAM was administered using computer-assisted personal interviews (CAPI), a method which had not been utilised in the validation of the measure. CAPI combines the benefits of face-to-face interviewing, such as encouraging longer responses to open-ended (unprompted) questions while minimising some of the disadvantages, such as social desirability bias and reluctance to disclose sensitive information [37]. Nevertheless, further research is needed to understand the impact of delivery mode on the reliability and validity of the Bowel/Colorectal CAM.

## Conclusions

The Bowel/Colorectal CAM meets accepted psychometric criteria for reliability and construct validity and should provide a useful tool for assessing CRC awareness, evaluating the effectiveness of education campaigns designed to improve awareness, and comparing across studies.

Public awareness of CRC symptoms and risk factors remains stubbornly low emphasising the importance of continuing to educate the public, particularly about the link between lifestyle behaviours and cancer risk. Communicating messages about cancer symptoms and risk factors is not easy because many cancer concepts and vocabulary are difficult to understand and people tend to get the gist but not the detail [38]. In addition, media messages can often be misleading [e.g. "Red wine can help prevent breast cancer" [39]] which adds further confusion. Efforts are needed to ensure that public health messages are consistent and coordinated and created using insights from the cancer communication literature. Awareness of symptoms and risk factors may be associated with more positive attitudes towards CRC and could also lead to increased engagement with screening [8], which may significantly reduce CRC incidence and mortality [5,40].

## Competing interests

The authors declare that they have no competing interests.

## Authors' contributions

EP participated in the design of study 2, carried out the analyses for study 2 and drafted the manuscript, AS participated in design of both studies, helped coordinate validation studies and assisted in drafting the manuscript, DJ participated in the design of study 1, collected data, carried out analyses and helped to draft study 1. SH assisted in drafting the manuscript. JW assisted in developing and provided final approval for the study designs and contributed to the drafting of the paper. All authors read and approved the final manuscript.

## Endnotes

**a**. The Bowel/Colorectal CAM can be downloaded from the Cancer Research UK website: http://tiny.cc/m7bn1

**b**. It is not appropriate to test unprompted items for internal reliability (Cronbach's alpha) or test-retest reliability.

**c**. 95% of CRC cases occur in those aged 50 and over [2].

## Pre-publication history

The pre-publication history for this paper can be accessed here:

http://www.biomedcentral.com/1471-2407/11/366/prepub

## Supplementary Material

Additional file 1 **Help-seeking item**. Click here for file

Additional file 2 **Lifetime risk question**. Click here for file
