# Supplementary material for: Assessing awareness of colorectal cancer symptoms: Measure development and results from a population survey in the UK
Source: BMC Cancer. 2011 Aug 23;11:366. doi: 10.1186/1471-2407-11-366 (PMC3188511; doi:10.1186/1471-2407-11-366)
Supplement: Additional file 1 — Help-seeking item. [file 1471-2407-11-366-S1.DOCX]

**Help-seeking item including response options**

| If you had a symptom that you thought might be a sign of bowel cancer how soon would you contact your doctor to make an appointment to discuss it? | | | | | | | | | |
| --- | --- | --- | --- | --- | --- | --- | --- | --- | --- |
| 1-3 days | 4-6 days | 1 week | 2 weeks | 1 month | 6 weeks | 3 months | 6 months | 12 months | Never |
|  |  |  |  |  |  |  |  |  |  |
