# Supplementary material for: Assessing awareness of colorectal cancer symptoms: Measure development and results from a population survey in the UK
Source: BMC Cancer. 2011 Aug 23;11:366. doi: 10.1186/1471-2407-11-366 (PMC3188511; doi:10.1186/1471-2407-11-366)
Supplement: Additional file 2 — Lifetime risk question. [file 1471-2407-11-366-S2.DOCX]

**Lifetime risk question**

| **Here is a picture of 100 people. Out of 100 people, how many do you think will develop bowel cancer at some point in their life?**      ___ ___ ___ **people out of 100 will develop bowel cancer at some point in their life** |
| --- |
